# Does knee joint cooling change in vivo patellar tendon mechanical properties?

**DOI:** 10.1007/s00421-016-3444-5

**Published:** 2016-07-29

**Authors:** Luis M. Alegre, Michael Hasler, Sebastian Wenger, Werner Nachbauer, Robert Csapo

**Affiliations:** 1 GENUD Toledo Research Group, University of Castilla-La Mancha, Avda. Carlos III s/n, 45071 Toledo, Spain; 2 Centre of Technology of Ski and Alpine Sport, Fürstenweg 187, Innsbruck, Austria; 3 Department of Sport Science, University of Innsbruck, Fürstenweg 185, Innsbruck, Austria

**Keywords:** Tendon deformation, Material properties, Ultrasonography, Cryotherapy

## Abstract

**Purpose:**

This study aimed to assess the influence of knee joint cooling on the in vivo mechanical properties of the patellar tendon.

**Methods:**

Twenty young, healthy women volunteered for the study. B-mode ultrasonography was used to record patellar tendon elongation during isometric ramp contraction of the knee extensors (5–7 s, 90° knee angle) and calculate tendon stiffness. Skin temperature was measured by infrared thermometry. Data were acquired before and after 30 min of local icing of the knee joint and compared by paired samples *t*-tests.

**Results:**

After cold exposure, skin temperature as measured over the patellar tendon dropped by 16.8 ± 2.0 °C. Tendon stiffness increased from 2189 ± 551 to 2705 ± 902 N mm^−1^ (+25 %, *p* = 0.007). Tendon strain decreased by 9 % (*p* = 0.004). A small, albeit significant reduction in maximum tendon force was observed (−3.3 %, *p* = 0.03).

**Conclusions:**

Knee cooling is associated with a significant increase in patellar tendon stiffness. The observed tendon stiffening may influence the operating range of sarcomeres, possibly limiting the maximal force generation capacity of knee extensor muscles. In addition, a stiffer tendon might benefit rate of force development, thus countering the loss in explosiveness typically described for cold muscles.

## Introduction

Connecting muscles to bone, tendons play an essential role in the transmission of contractile force as well as the storage and recovery of elastic energy during locomotion. Their mechanical and material properties, which are typically expressed by their stiffness and Young’s modulus, strongly influence the function of muscle–tendon units (Alexander 2002). Stiffer tendons facilitate accurate joint position control and a more rapid rate of force development, whereas more compliant tendons are commonly assumed to benefit movement economy in cyclic motions (Biewener and Roberts 2000; Kubo et al. 2010), although recent findings have challenged this notion (Albracht and Arampatzis 2013). Tendon compliance also affects a muscle’s length–tension and force–velocity relationship and, thus, the maximum force that it can generate in both static and dynamic contractions. Considering their functional importance, several attempts have been made to alter tendon stiffness through long-term training interventions. For instance, resistance training has been found to be effective in reversing the loss in tendon stiffness and quality observed at older age (Reeves et al. 2003). Conversely, continuous stretching interventions may lower tendon stiffness to benefit joint range of movement (Konrad et al. 2015).

In preparation for physical activity or rehabilitation, it is sometimes also desirable to acutely influence the elastic properties of tendons and other collagenous tissues. As an example, acute stretching, which may induce a transient decrease in tendon stiffness (Obst et al. 2013), is frequently incorporated into warm-up routines to reduce the likeliness of musculo-tendinous injuries (Woods et al. 2007). In physiotherapeutic settings, thermal agents are often used to modify the viscoelastic properties of collagens (Bleakley and Costello 2013). However, scant basic research exists to directly test the effects of changes in temperature on tendon mechanics. In vitro studies suggest that tendon stiffness increases and viscous mechanical behavior decreases at lower temperatures (Huang et al. 2009; Wang et al. 2005), but the necessary preservation of tendons as well as the clamping of specimen during tensile tests may complicate the extrapolation of results to in vivo conditions (Maganaris et al. 2004). The only two studies to use imaging techniques to investigate temperature-associated changes in tendon stiffness in humans in vivo have shown equivocal results. While Kubo et al. (2005a) found the stiffness of the Achilles tendon to be unaffected by both hot and cold water immersion, Muraoka et al. (2008) reported a significant, albeit small increase in stiffness after cooling the lower leg by ~6 °C. No studies performed to date have tested the effects of temperature on the stiffness and material properties of the human patellar tendon. The deliberate use of heat or cold to modify tendon mechanical properties represents a novel concept that may be of great relevance in both sports and rehabilitation. If tendons were indeed found to be sensitive to changes in temperature, the application of thermal agents might be used to tailor tendon stiffness for optimized athletic performance or in preparation for subsequent physiotherapy.

In the light of these considerations, the present study aimed to investigate the consequences of local cooling of the knee on the in vivo mechanical properties of the human patellar tendon. Using a repeated measure study design, we studied a sample of healthy female subjects before and after application of an ice pack placed on top of the patellar tendon. Based on the results of in vitro studies, we hypothesized that cooling would lead to a stiffening of the patellar tendon through an equivalent increase in Young’s modulus.

## Methods

### Study design

To assess the influence of temperature on the in vivo patellar tendon mechanical properties, the force–elongation relationship was assessed by ultrasonography and dynamometry before and after 30 min of cooling through application of an ice pack (a 1-l ziploc bag filled with snow) placed directly on the patellar tendon. Room temperature was set at 22 °C at a relative humidity of 40 %, and kept constant during the whole protocol. The ultrasound gel used for image acquisition was also kept at room temperature. Skin temperature over the patellar tendon was recorded by infrared thermometry (TFA 31.1108, TFA Dostmann, Wertheim, Germany) at six different points of time: (1) before tendon warm up-conditioning; (2) after warm up-conditioning, just before testing trials; (3) after testing trials, just before cold exposure; (4) immediately after cold exposure, before post-cooling warm up; (5) after post-cooling tendon warm up-conditioning, just before testing trials; and (6) after the post-cooling testing trials.

### Subjects

Twenty healthy young women, all of them university students or members of the staff of the local department (24.8 ± 4.2 years, 166 ± 6 cm and 59.9 ± 7.1 kg) volunteered for the study. This sample size was determined by an a priori power analysis (*α* = 0.05, 1 − *β* = 0.80, dz = 0.60), based on changes in tendon stiffness observed in a pilot study, carried out on six members of the staff of the department (5 men and 1 woman, age: 26.3 ± 3.3 years; stiffness values: pre-cooling 2512 ± 741 vs. post-cooling 2987 ± 835 N mm^−1^). Exclusion criteria for participation in the experiment were the occurrence of serious lower limb injuries in the past 2 years as well as any health conditions precluding maximal strength testing. Before giving written consent, the participants were informed about the design and potential risks of the study as well as possible discomfort related to measurements. The study was approved by the local Institutional Review Board (vote 26/2015) and conducted in agreement with the ethical principles for medical research outlined in the Declaration of Helsinki (World Medical Association 2013).

### Measurement of tendon elongation

The whole testing procedure was performed on the dominant leg of all participants. Resting patellar tendon length was measured by B-mode ultrasonography (MyLab25, Esaote, Genoa, Italy; probe: LA523, 50 mm, 7.5- to 12-MHz transducer; axial and lateral spatial resolution: 0.12 and 0.14 mm, respectively) as the distance between the patellar apex and the tibial tuberosity. For this purpose, a panoramic image was generated in the sagittal plane using the scanner’s built-in pattern-matching technology, while subjects held their knees flexed at 90°. Tendon cross sectional area (CSA) was measured from a single cross sectional image obtained at mid distance between the patella and the tibial tuberosity, with the knee fully extended. Intra-day reliability was assessed in a subgroup of ten subjects. Typical errors (Hopkins 2000) were 1.6 % (1.25 mm^2^) for patellar tendon CSA and 2.3 % (1.0 mm) for patellar tendon length.

**Fig. 1:**
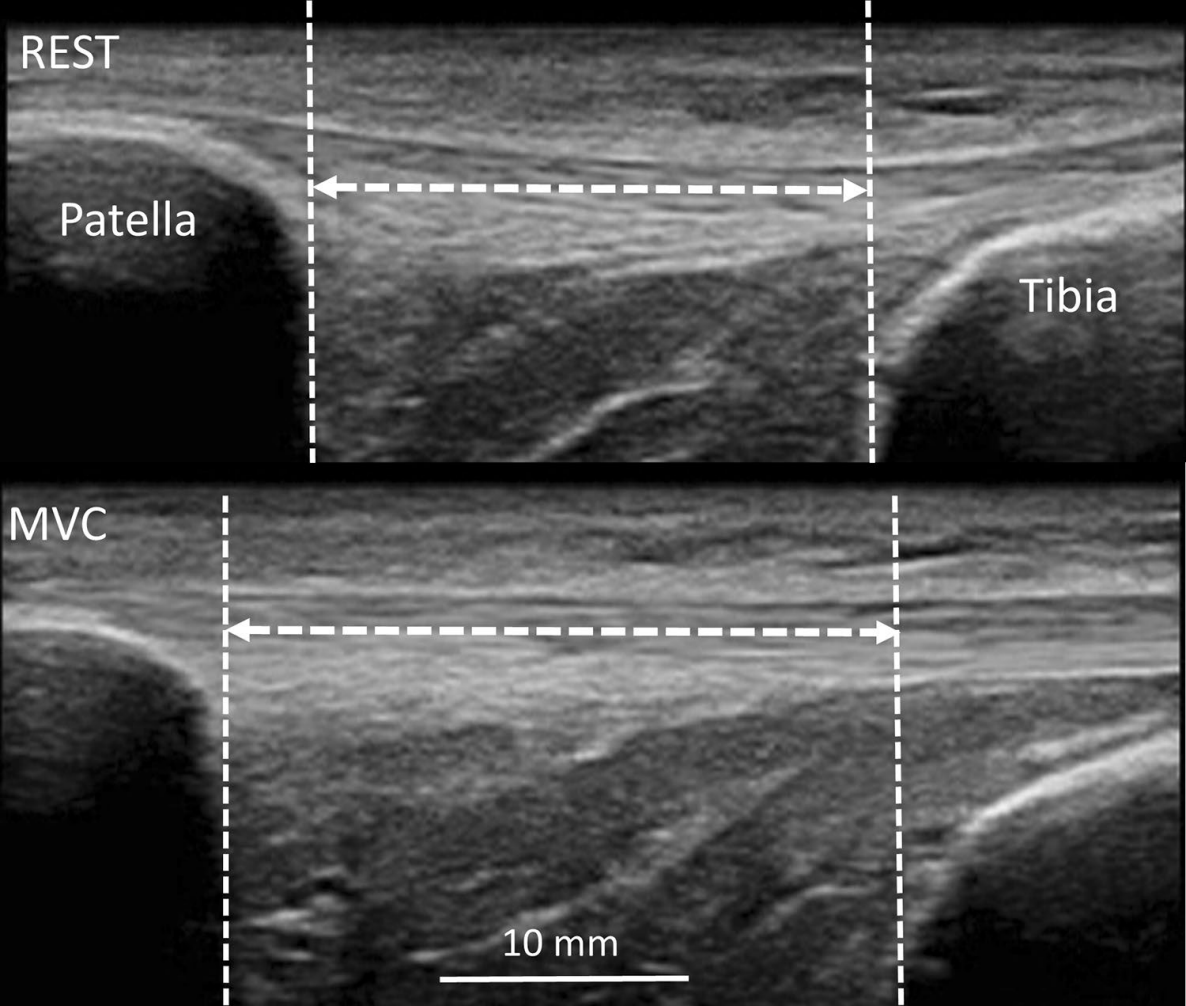
Measurement of patellar tendon elongation on the ultrasound images recorded during one maximal voluntary isometric contraction (MVC)

For measurements of maximum voluntary contraction strength (MVC) and tendon mechanical properties, participants were seated on a custom-built examination bed with their legs dangling over its edge. A standard load cell (Wägezelle U2A, HBM, Austria) was fixed to a horizontal bar mounted onto the rear end of the bed at a height equivalent to that of the ankle malleoli. A steel cable and a leather ankle cuff were used to connect the load cell to the lower leg just proximal of the malleoli. The length of the steel cable was adjusted to fix the knee at 90° during MVC (0° representing full extension). During contractions, participants were instructed to grab the frame of the bed to help stabilize their pelvis and upper body. After familiarization with the testing protocol, which consisted of several submaximal isometric ramp contractions with visual feedback and constant loading rate, the participants performed a warm up of five to seven submaximal ramp contractions and two additional maximal contractions, which also served for tendon conditioning. Then, using the same linear array ultrasound transducer utilized for the assessment of tendon morphology, images of the patellar tendon were obtained in the sagittal plane, such that both the patella and the tibial tuberosity were in the field of view. With the probe held firmly in place, ultrasound video sequences were recorded while the participants performed between five and seven maximal ramp contractions at a constant, standardized loading rate. One minute of passive recovery was granted between contractions. Trials yielding inadequate image quality were discarded and repeated. Tendon elongation was measured offline as the proximodistal component of the displacement of the patellar apex relative to the tibial plateau (Fig. 1). For this purpose, specific software facilitating semi-automated tracking (Tracker 4.91, http://physlets.org/tracker/) of points of interest was used. This approach allowed us to obtain continuous force–elongation data (rather than discrete values only) for the subsequent calculations of tendon material properties. Force (100 Hz) and video (24 Hz) data were synchronized with a custom-built trigger that generated an electrical pulse that was simultaneously recorded through the scanner’s ECG input and by an external 12-bit A/D converter (Spider8, HBM, Austria). All force and elongation data were processed offline using custom-made MATLAB^®^ routines (MATLAB R2014b, Mathworks, Natick, MA, USA). Knee extension torque was calculated as the product of knee extension force and the distance between the center of rotation of the knee and the point of attachment of the cable at the ankle cuff. Then, this product was divided by the individual patellar tendon moment arm, estimated from femoral length (Visser et al. 1990), to obtain tendon force.

The trials with the lowest and greatest tendon elongation from each condition were excluded from analysis. Force–elongation curves were either fitted with second or third order polynomials (*r*^2^ > 0.97), but fitting techniques were kept constant within subjects, under both warm and cold conditions. To compare each individual’s tendon mechanical properties at similar force levels, stiffness was measured at the maximum 10 % of force achieved during the weakest valid trial. Young’s modulus was then estimated by multiplying stiffness with the tendon resting length over CSA ratio. Stress and strain were obtained by dividing maximum tendon force by CSA and maximal tendon elongation by resting length, respectively. Tendon dimensions are not expected to change in response to cooling and, consequentially, temperature-associated changes in stiffness and Young’s modulus are equivalent. Therefore, CSA and resting length were acquired prior to cooling, and Young’s modulus and stress values were reported for the baseline (warm) condition only. Care was taken to assure similar loading rates (i.e., force–time curves) between warm and cold conditions (+1 % difference between conditions, *t*(19) = 0.791, *p* = 0.439) since this factor may influence the assessment of tendon mechanical properties (Kosters et al. 2014). Typical errors, computed from intra-day test–retest trials, were 6.9 % (163 N mm^−1^) for tendon stiffness, 6.9 % (0.1 GPa) for Young’s modulus, 4.3 % (0.175 mm) for tendon elongation, 4.4 % (0.4 %) for maximal strain, and 2.5 % (139 N) for maximal tendon force, respectively.

### Data analysis

Statistical analysis was performed with SPSS Version 22 (SPSS Inc., IBM, USA). Data are presented as means ± SDs. Normality of all variables was tested by Shapiro–Wilk tests. The time course of changes in skin temperature at the patellar tendon was assessed by a one-way, repeated measure ANOVA, and Bonferroni-adjusted *t*-tests were used for post hoc pairwise comparisons. Since significant Mauchly’s tests indicated a violation of the assumption of sphericity,* F*-values were adjusted using the Greenhouse–Geisser correction (*ε* < 0.75). Changes in tendon mechanical properties and tendon force were assessed with *t*-tests for paired samples. Results were considered significant at *p* < 0.05. Mean effect sizes (ES) were calculated to quantify the dimension of difference between warm and cold conditions (Cohen 1988). ES of 0.2, 0.5 and 0.8 were interpreted as small, moderate, and large effects, respectively.

## Results

**Fig. 2:**
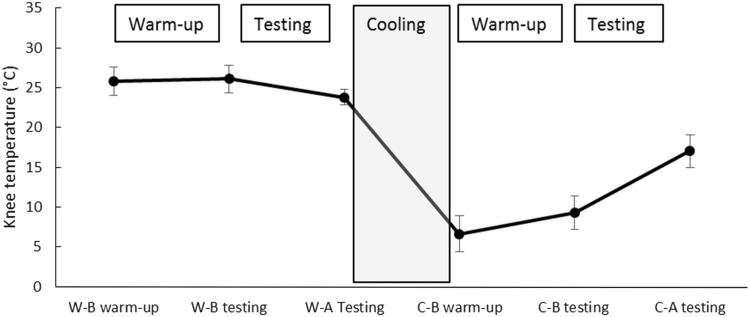
Changes in skin temperature over the patellar tendon throughout the experiment. *W-B* warm before, *W-A* warm after, *C-B* cold before, *C-A* cold after

The cooling intervention significantly decreased skin temperature over the patellar tendon (*F*(2.26, 45.12) = 989.48, *p* < 0.001) (Fig. 2). Specifically, temperature dropped from 23.8 ± 1.0 °C (after the testing trials in the “warm” condition) to 6.6 ± 2.3 °C (immediately after cold exposure; *p* < 0.001). Likewise, a significant difference for skin temperature was found when comparing the measurement values obtained just before the beginning of testing trials (26.1 ± 1.7 °C prior to cooling vs. 9.3 ± 2.1 °C after ice application, *p* < 0.001).

### Tendon morphology and material properties

Patellar tendon CSA and resting length were 75.5 ± 11.9 mm^2^ and 45.0 ± 3.9 mm, respectively. In the warm condition, Young’s modulus and maximum stress were 1.32 ± 0.34 GPa and 69.2 ± 17.7 MPa, respectively. After 30 min of knee cooling, patellar tendon stiffness increased significantly from 2189 ± 551 to 2705 ± 902 N mm^−1^ (+25.4 ± 36.6 %, *t*(19) = −3.046, *p* < 0.007, ES = 0.69). Maximal strains measured before and after cooling of the knee joint were 8.6 ± 1.6 % (3.89 ± 0.96 mm) and 7.8 ± 1.4 % (3.50 ± 0.76 mm), respectively (−9.1 ± 11.6 %, *t*(19) = 3.230, *p* = 0.004, ES = 0.55). Also, maximal tendon forces were found to decrease from 5148 ± 1176 to 4957 ± 1048 N in response to the cooling intervention (−3 ± 6.9 %, *t*(19) = 2.348, *p* = 0.030), although Cohen’s *d* reflected a small effect size (ES = 0.17 for tendon forces).

**Fig. 3:**
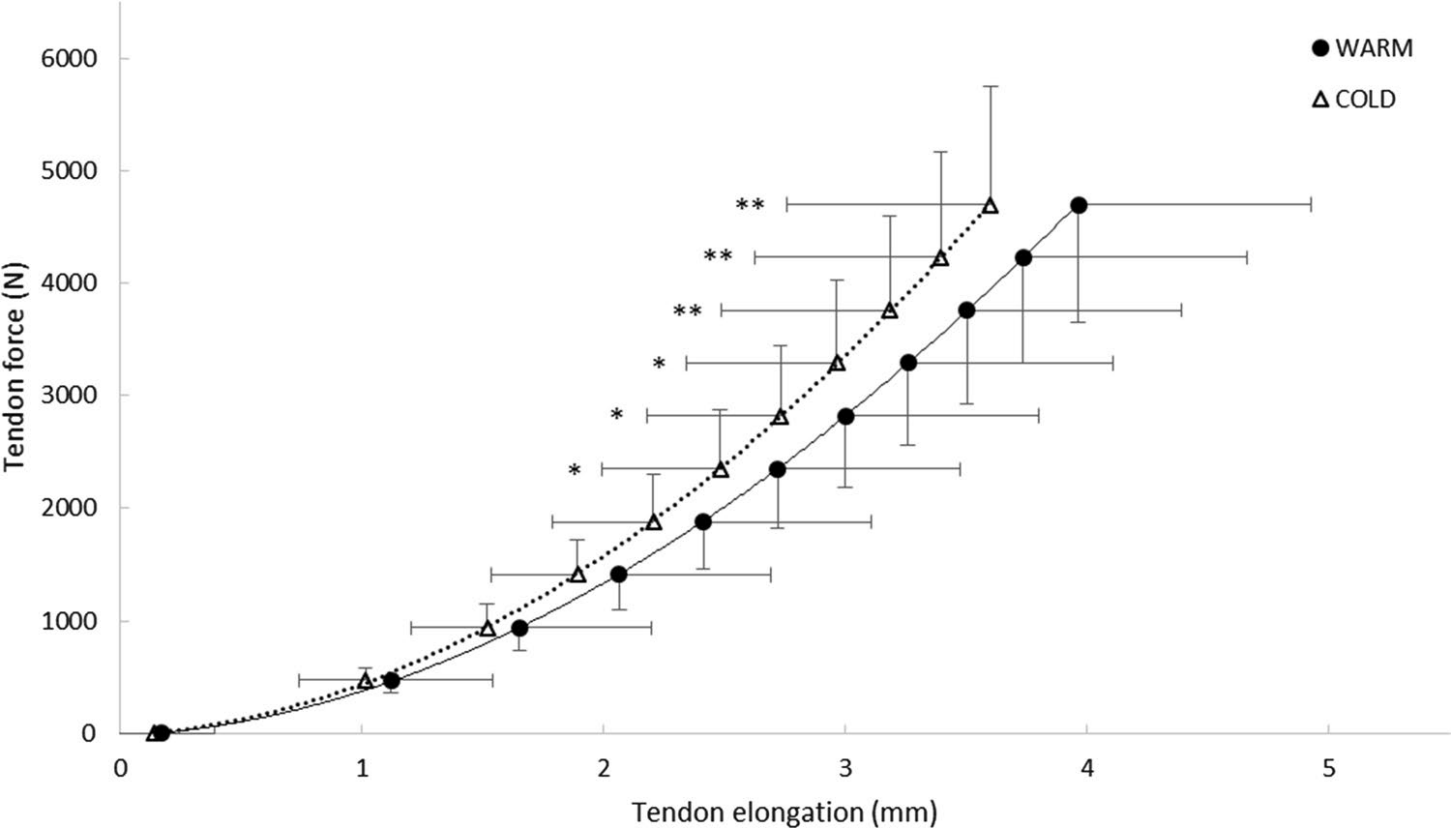
Force-elongation curves as measured under warm and cold conditions. **p < 0.01; *p < 0.05

Temperature-associated differences in tendon elongation were significant between 50–100 % of maximal tendon force, as evidenced by the force–elongation curves shown in Fig. 3. Effect sizes revealed that temperature-related differences in tendon elongation (ES = 0.38–0.40) increased gradually in this force range.

## Discussion

The current investigation aimed to assess the effects of knee cooling on the mechanical properties of the patellar tendon, to better understand the effects of cryotherapy on muscle–tendon unit function. Our findings showed significant increases in tendon stiffness (+25 %, *p* = 0.007, ES = 0.69), accompanied by decreases in maximal strain (−9 %, *p* = 0.004, ES = 0.55). Maximum force output was slightly but significantly affected, with a −3.3 % decrease in MVC seen after cooling (*p* = 0.03, ES = 0.17).

In our study, 30 min of ice application resulted in an average decrease of skin temperature of ~17 °C (Fig. 2). Dewhurst et al. (2010b) used between 20 and 35 min of cooling with ice bags to decrease thigh skin temperature by ~6 °C. At the same time, vastus lateralis muscle temperature, as measured 1 cm below each subject’s subcutaneous fat layer, decreased by 4 °C. In another study, Warren et al. (2004) also used ice bags for 30 min to decrease knee intraarticular temperature by 3.3 °C (at the suprapatellar pouch), with a concomitant skin temperature decrease of 21 °C. While no studies exist to directly assess the effects of knee joint cooling on patellar tendon temperature, Selkow et al. (2014) reported that 20 min of ice bag cooling reduced the Achilles tendon’s internal temperature from 29.3 to 16.7 °C. Of note, skin temperature values reported in this study (27.7 and 7.1 °C) were very similar to those measured here at equivalent points of time (26.1 °C prior to cooling vs. 9.3 °C after ice application). Although the extrapolation of changes in skin temperature to those that occur at the depth of the patellar tendon is complicated, it is worth noting that the patellar tendon lies close to the skin surface (~5 mm depth in most of our subjects), has a limited blood supply (Józsa and Kannus 1997) and is not insulated by highly perfused organs, such as skeletal muscles. Considering the significant decrease in skin temperature, it is plausible to assume that our cooling intervention elicited a drop in tendon temperature that is of greater dimension as that reported for muscle and joint in the studies by Dewhurst et al. (2010a) and Warren et al. (2004), and similar to that reported for the Achilles tendon by Selkow et al. (2014). As a predominantly collagenous tissue, tendon is also known to be very sensitive to changes in temperature (Chan et al. 1998; Ciccone et al. 2006; Huang et al. 2009; Wang et al. 2005), so we are confident that our cooling protocol elicited a strong enough stimulus to alter its mechanical properties.

In vitro studies (Ciccone et al. 2006; Huang et al. 2009; Noonan et al. 1993; Wang et al. 2005) have demonstrated an inverse relationship between connective tissue temperature and stiffness. However, results of ultrasound-based human in vivo studies are controversial (Kubo et al. 2005b; Muraoka et al. 2008), which may partly be explained by methodological differences (Muraoka et al. 2008). While these two investigations focused on the aponeurosis and tendon of the gastrocnemius muscle, the present study is the first to apply imaging techniques to study the temperature-dependency of the patellar tendon’s mechanical properties. It is worth mentioning that we made a particular effort to simultaneously scan both the proximal and distal tendon attachments, which is currently considered the best approach to analyze the patellar tendon mechanical properties by B-mode ultrasonography (Seynnes et al. 2015). Supporting the results of in vitro studies and in agreement with our hypothesis, we found the ice application to provoke a substantial increase in tendon stiffness (+25 %).

Our tests do not allow to ascertain the mechanisms underlying the increase in tendon stiffness. Theoretically, the major contributors to increased tensile strength are a larger cross-sectional area (Kongsgaard et al. 2007), increased collagen content (Woo et al. 1980) and cross-linking (Fessel et al. 2012) as well as alterations in the structural arrangement of collagen fibrils (Fratzl 2008). However, a short-term cooling intervention, leading to a transient increase in tendon stiffness, would not alter any of these parameters. A more plausible explanation for the increased tendon stiffness may lie in changes of the material properties of collagen fibrils. Recent research by Gevorkian and colleagues (2009, 2013) has demonstrated that the collagen triple-helix constituting the microfibrils of collagen type I fibers (i.e., the by far most prevalent type of collagen in tendons) is thermally unstable at physiological temperatures. Studying collagenous material obtained from rat Achilles tendons, the authors found the Young’s modulus of collagen fibrils to be higher by ~14 % at 20 °C as compared to 40 °C (Gevorkian et al. 2009). If we assume that our cooling intervention elicited changes in patellar tendon temperature that are of comparable dimension as those reported by Selkow et al. (2014) for the Achilles tendon (i.e., a decrease from ~29 to ~17 °C), and if we further take the methodological differences between our study and that of Gevorkian et al. (2009) into account (in vivo vs. in vitro, tendon force estimation vs. direct force measurement, etc.), it is plausible to assume that our findings represent the in vivo equivalence of Gevorkian’s results. Thus, cooling-associated increases in tendon stiffness might be related to changes in the intrinsic material properties of collagen fibrils. Yet another consequence of tendon cooling might be a change of the viscosity of the tendon’s gel-like ground substance (Józsa and Kannus 1997). Further research is warranted to assess potential temperature-associated changes in the viscoelastic properties of patellar tendon, and their functional consequences.

The stiffening of a tendon has numerous important functional implications. First and foremost, tendon stiffness strongly influences the maximum amount of force a muscle can generate at a given joint position, as may be exemplified based on our observation of a small but significant (−3.3 %, ES = 0.17, *p* = 0.030) decrease in MVC force after cooling of the knee. This finding was unexpected because previous studies to investigate the consequences of muscle cooling either found isometric force production to have a low temperature dependency (Thornley et al. 2003; Drinkwater 2008a), or even reported slight increases in muscle strength after cooling (Ranatunga et al. 1987). In addition, it needs to be stressed that, in the present study, we made a particular effort to cool the knee joint only, while no intervention was applied at the muscle level. To understand the observed decrease in MVC force, the role the tendon plays in setting sarcomere operating range needs to be considered. The stiffness of a tendon determines the degree by which a muscle’s sarcomeres are allowed to shorten upon contraction. To exemplify this concept, we estimated the degree of vastus lateralis sarcomere shortening by dividing fascicle shortening (~40 mm during maximal isometric contractions: cf. Ichinose et al. (1997)) by the anticipated average number of sarcomeres in series (average resting fascicle length ~100 mm: cf. Ichinose et al. 1997; sarcomere length ~3.2 µm: cf. Cutts 1988). Considering that tendon strain was lower by ~10 %, maximal sarcomere shortening would be reduced by ~0.2 µm after cooling of the knee joint. Assuming that at a 90° knee angle the sarcomeres of the quadriceps femoris muscle would be operating on the descending limb of the length–tension curve (Ando et al. 2016; Reeves et al. 2004), a stiffer tendon would impede muscular shortening more, force sarcomeres to operate farther to the right (i.e., lower) on their length–tension relationship and, thus, contribute to the observed reduction in force output. In fact, a right-shift of 0.2 µm on the sarcomeres’ length–tension curve of is equivalent to a ~8 % decrease in tension (Cutts 1988). Possibly, at shorter muscle lengths, where sarcomeres might operate on the ascending limb of the length–tension relationship, the increased tendon stiffness would have resulted in an increase of MVC strength. However, this latter statement is speculative and warrants further research.

Yet another characteristic of muscle function that is influenced by tendon mechanical properties is rate of force development (RFD). While not measured in this study, RFD is known to be positively correlated to tendon stiffness (Bojsen-Moller et al. 2005). This is because a stiffer elastic element placed in series with the muscle allows for a more rapid transmission of force from muscle to bone. In a more realistic scenario, characterized by a decrease of temperature in the whole muscle–tendon unit (as may occur in outdoor sports under cold environmental conditions), an increase in tendon stiffness might act to counter the expected decrease in contractile RFD, resulting from cooling of the muscle (Drinkwater 2008b). A stiffer tendon would also facilitate more accurate control of joint position (Rack and Ross 1984). Paradoxically, it might at the same time also hinder force control because the dampening effect of a compliant tendon, that may absorb random deviations in muscle force output, would be limited. Future studies to be carried out in our laboratory will test whether the simultaneous application of two interventions to warm muscles (to increase the rate of force generation) and cool tendons (for improved force transmission) would lead to actual increases in RFD and performance in explosive athletic tasks.

**Fig. 4:**
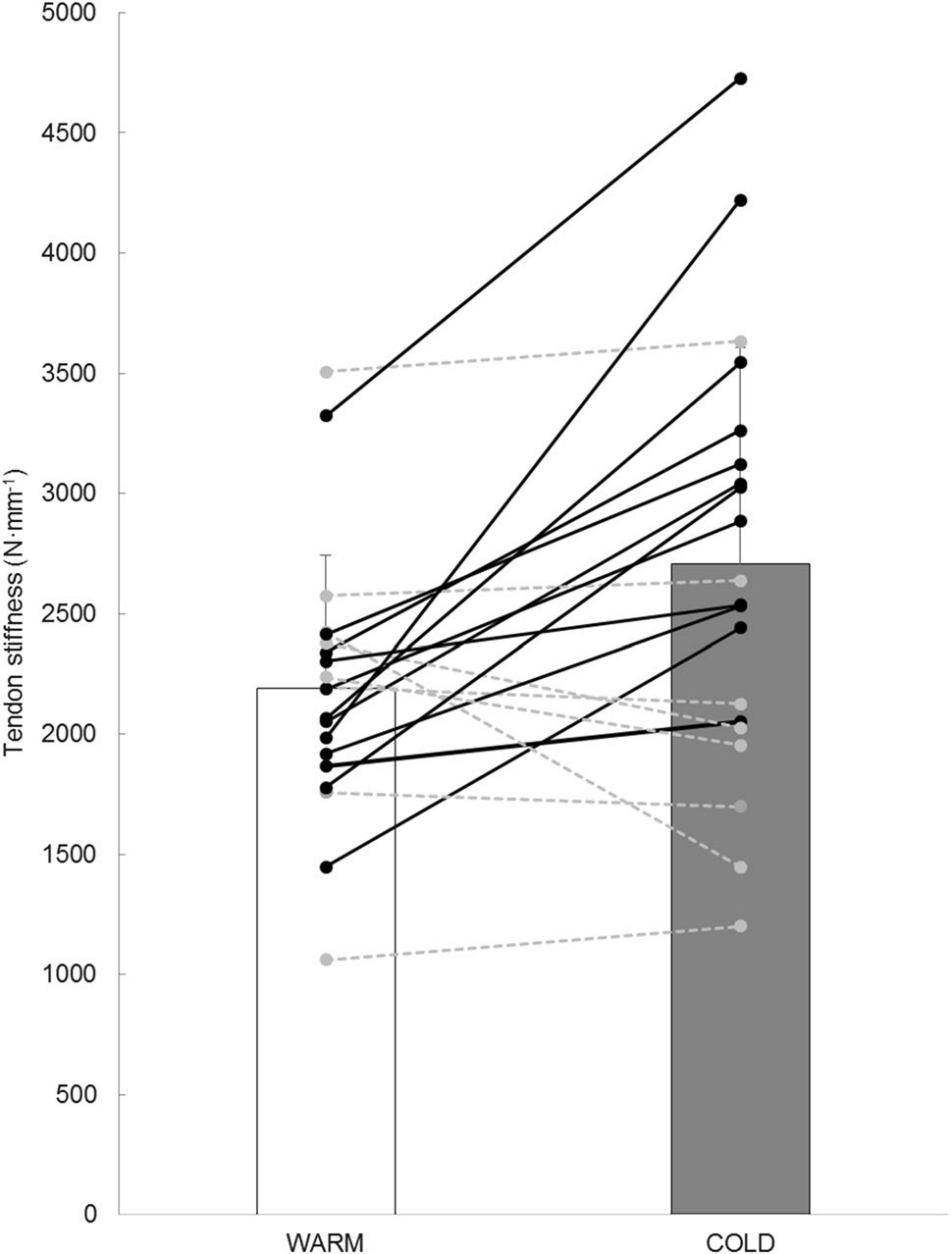
Individual values of patellar tendon stiffness as measured before and after knee cooling. Subjects showing increases greater than the typical measurement error (163 N mm^−1^) are shown in *black*, *solid lines*, while others are shown in *grey*, *dashed lines*. *Bars* show means ± SD

Similar to other studies performed with ultrasonography (Muraoka et al. 2008), we observed large inter-individual variability in the changes in stiffness associated with cooling (mean change of 25.4 ± 36.6 %, individual responses shown in Fig. 4). Fifteen out of 20 subjects showed increases in stiffness, and 12 of them above the typical error computed in our pilot experiment (163 N mm^−1^). It should be noted that even if we removed the value of one outlier (+113 % in tendon stiffness after knee cooling), temperature-associated changes in tendon stiffness would still be significant: +20.5 ± 31.1 %, *p* = 0.011; ES = 0.59. This points out to the existence of “responders” who would react more strongly to acute cold exposures. While elucidation of the reasons responsible for these differential responses to our cooling intervention goes beyond the scope of this study, it is possible that collagen fibrils with different intrinsic material properties (Wenger et al. 2007) would react more or less sensitively to temperature variations. Also, differences in subcutaneous adipose tissue thickness could have played a role, with thicker fat layers providing better insulation of the tendon and, therefore, decreasing its thermal sensitivity. Unfortunately, adipose layer thickness could not be reliably determined from our ultrasound scans because the contact pressure required to hold the probe in place during muscular contraction was associated with inevitable compression of skin and subcutaneous fat. Apart from biological explanations, it is important to note that, in spite of our best efforts to optimize reliability, parts of the variability reported in our findings may be related to inherent measurement inaccuracies, present in the vast majority of the current studies using ultrasound-based assessment of in vivo tendon mechanical properties. Still, the present study was carefully designed, trying to control or limit these methodological limitations:

- The reliability and validity of B-mode ultrasound for the measurement of patellar tendon CSA has been challenged (Ekizos et al. 2013). While the validity of ultrasound was not directly tested in this study, our results agree with those obtained in other recent publications to show good reproducibility of tendon CSA measurements (Rieder et al. 2015). More importantly, it should be noted that values of tendon CSA and, consequently, Young’s modulus were only reported for descriptive purposes, so potential inaccuracies would have no direct impact on our main findings.
- Also, it should be noted that probe size (50 mm) may be a limiting factor when tracking tendon elongation in particularly tall subjects. However, our tallest participant was only 177 cm and the two reference points (the patella and the tibial tuberosity) are less apart than the tendon’s most distal and proximal attachment sites. Therefore, the reference points for tendon tracking were clearly within the field of view in all included trials and this method is accepted as valid in the current scientific literature (Kosters et al. 2014; Rieder et al. 2016; Wiesinger et al. 2016).
- Errors in the calculation of joint moments due to knee rotation during MVC trials could have led to inaccuracies in the tendon force calculations, which have been found to amount to up to 17 % (Arampatzis et al. 2004; Tsaopoulos et al. 2011). However, we (1) used a very rigid seat for dynamometric measurements to limit contraction-associated changes in knee joint position, and (2) ensured that joint position was constant during MVC. In addition, it must be stressed that our study relied on within-subject comparisons, so we expect possible errors in the tendon force calculations between the warm and cold condition to be small.
- Differences in tendon elongation at 100 % of common force levels were small (~0.4 mm) and could, therefore, have been difficult to detect. Nonetheless, it should be noted that the spatial resolution of our ultrasound scanner is 0.12–0.14 mm/pixel, and the typical error of this parameter was calculated to be 0.18 mm, that is, well below the mean change reported. Also, our analyses suggest that the tendon stiffening observed in our study was beyond reasonable statistical doubt.
- Although we did not measure agonistic and antagonistic electromyographic activity and its effects on tendon force, it is worthy to note that our cooling intervention was only applied on the knee joint, with constant environmental temperature throughout the testing protocol. Therefore, changes in antagonistic co-activation (that could have affected the net force output of agonist muscles) are very unlikely.
- Overestimation of tendon elongation from rotational movements of the patella and tibia during contraction (Seynnes et al. 2015) was controlled by measuring tendon elongation from the proximodistal component of the displacement of the patellar apex relative to the tibial plateau.
- While patellar tendon moment arm length was estimated from height rather than directly measured, this parameter was constant (within-subject comparisons) and could not affect our central outcome measures.
- Finally, warm-up and loading rate during the testing contractions, which have been reported to significantly affect tendon material properties (Kosters et al. 2014), were also kept constant between experimental conditions, to control potential bias from these two sources.

In conclusion, 30 min of joint cooling increased patellar tendon stiffness, probably through changes in the material properties of collagen fibrils. This change is presumed to affect muscle tendon unit function, by altering the operating range of quadriceps sarcomeres on their length–tension relationship and increasing rate of force development in explosive contractions. Further research is warranted to analyze the effects of different temperature combinations on specific components of the muscle–tendon unit.

## Abbreviations

ANOVA: Analysis of variance
CSA: Cross sectional area
ES: Effect size
MVC: Maximum voluntary contraction
RFD: Rate of force development
